# Human lung myofibroblast TGFβ1-dependent Smad2/3 signalling is Ca^2+^-dependent and regulated by K_Ca_3.1 K^+^ channels

**DOI:** 10.1186/s13069-015-0022-0

**Published:** 2015-03-26

**Authors:** Katy M Roach, Carol Feghali-Bostwick, Heike Wulff, Yassine Amrani, Peter Bradding

**Affiliations:** Department of Infection, Immunity and Inflammation, Institute for Lung Health, University of Leicester, Glenfield Hospital, Groby Road, Leicester, LE3 9QP UK; Department of Medicine, Division of Rheumatology and Immunology, University of South Carolina, Columbia, SC 29208 USA; Department of Pharmacology, University of California, 451 Health Sciences Drive, Davis, CA 95616 USA

**Keywords:** Human lung myofibroblast, Idiopathic pulmonary fibrosis, Potassium channel KCa3.1

## Abstract

**Background:**

Idiopathic pulmonary fibrosis (IPF) is a common and invariably lethal interstitial lung disease with poorly effective therapy. Blockade of the K^+^ channel K_Ca_3.1 reduces constitutive α-SMA and Smad2/3 nuclear translocation in IPF-derived human lung myofibroblasts (HLMFs), and inhibits several transforming growth factor beta 1 (TGFβ1)-dependent cell processes. We hypothesized that K_Ca_3.1-dependent cell processes also regulate the TGFβ1-dependent Smad2/3 signalling pathway in HLMFs. HLMFs obtained from non-fibrotic controls (NFC) and IPF lungs were grown *in vitro* and examined for αSMA expression by immunofluorescence, RT-PCR, and flow cytometry. Two specific and distinct K_Ca_3.1 blockers (TRAM-34 200 nM and ICA-17043 [Senicapoc] 100 nM) were used to determine their effects on TGFβ1-dependent signalling. Expression of phosphorylated and total Smad2/3 following TGFβ1 stimulation was determined by Western blot and Smad2/3 nuclear translocation by immunofluorescence.

**Results:**

K_Ca_3.1 block attenuated TGFβ1-dependent Smad2/3 phosphorylation and nuclear translocation, and this was mimicked by lowering the extracellular Ca^2+^ concentration. K_Ca_3.1 block also inhibited Smad2/3-dependent gene transcription (αSMA, collagen type I), inhibited K_Ca_3.1 mRNA expression, and attenuated TGFβ1-dependent αSMA protein expression.

**Conclusions:**

K_Ca_3.1 activity regulates TGFβ1-dependent effects in NFC- and IPF-derived primary HLMFs through the regulation of the TGFβ1/Smad signalling pathway, with promotion of downstream gene transcription and protein expression. K_Ca_3.1 blockers may offer a novel approach to treating IPF.

**Electronic supplementary material:**

The online version of this article (doi:10.1186/s13069-015-0022-0) contains supplementary material, which is available to authorized users.

## Background

Idiopathic pulmonary fibrosis (IPF) is a common disease that occurs primarily in older people with a prevalence in the USA of 227 cases per 100,000 in the >75 year age group [1,2]. In the UK, its incidence doubled between 1998 and 2003 with 4,000 new cases per year, and the incidence continues to increase by 10% annually [2,3]. It therefore represents an important cause of morbidity and mortality, and current treatments such as pirfenidone are of limited efficacy [4-6].

Myofibroblasts are an attractive target for the treatment of IPF. They are the primary effector of tissue fibrosis as they synthesize large quantities of collagen [1], have a contractile phenotype [7-9] and are resistant to apoptosis [10]. Myofibroblast expansion occurs through the differentiation of fibroblasts [11]; this involves reorganization of the actin cytoskeleton, increased expression of alpha smooth muscle actin (αSMA) and incorporation of actin stress fibers [8]. As myofibroblasts rarely persist in healthy lungs, their differentiation is considered a key event in the pathogenesis of IPF.

Transforming growth factor beta 1 (TGFβ1) is a key pro-fibrotic growth factor involved in the pathogenesis of IPF [12-14], which stimulates fibroblast to myofibroblast differentiation both *in vivo* and *in vitro* [15-18]. The major TGFβ1-dependent signalling pathway involves the cytoplasmic Smad proteins [19]. Activated TGFβ1 binds to the TGFβ type II receptor (TGFβRII) leading to the recruitment of the TGFβ type I receptor, which induces the phosphorylation of the downstream targets Smad2 and Smad3. Phosphorylated Smad2 and Smad3 then form hetero-oligomeric complexes with Smad4 and translocate to the nucleus to regulate gene expression through transcription and thus mediate the biological effects of TGFβ1 such as cell growth, differentiation and contraction [20-22]. In particular, this TGFβ1/Smad pathway contributes to myofibroblast differentiation by increasing α-smooth muscle actin expression [20,21].

Ion channels are attractive therapeutic targets in many chronic diseases. In particular, the Ca^2+^ activated K^+^ channel K_Ca_3.1 plays an important role in Ca^2+^ signalling through its ability to maintain a negative membrane potential during cellular activation, which enhances Ca^2+^ influx from the extracellular fluid [23-25]. The K_Ca_3.1 channel modulates the activity of several structural and inflammatory cells [26-28], but more specifically, K_Ca_3.1 blockade inhibits several TGFβ1-dependent cell processes in primary human lung myofibroblasts (HLMFs) derived from both non-fibrotic and IPF lung tissue [29]. Our previous work demonstrated that blocking K_Ca_3.1 with the selective K_Ca_3.1 blockers TRAM-34 and ICA-17043 inhibits TGFβ1-dependent HLMF wound healing, collagen secretion, contraction and Ca^2+^ influx [29]. In addition, IPF-derived HLMFs demonstrate increased constitutive αSMA expression and Smad2/3 nuclear localisation, effects which are reversed by K_Ca_3.1 inhibition [30].

We hypothesized that K_Ca_3.1 channel activity also regulates TGFβ1-dependent responses in HLMFs through the TGFβ1/Smad signalling pathway. We therefore investigated the role of the K_Ca_3.1 channel in TGFβ1-dependent Smad2/3 phosphorylation, Smad2/3 nuclear translocation, gene transcription and αSMA protein expression in HLMFs obtained from both healthy and IPF lung.

## Results

### TGFβ1-dependent Smad2/3 phosphorylation is attenuated by K_Ca_3.1 blockade

**Figure 1:**
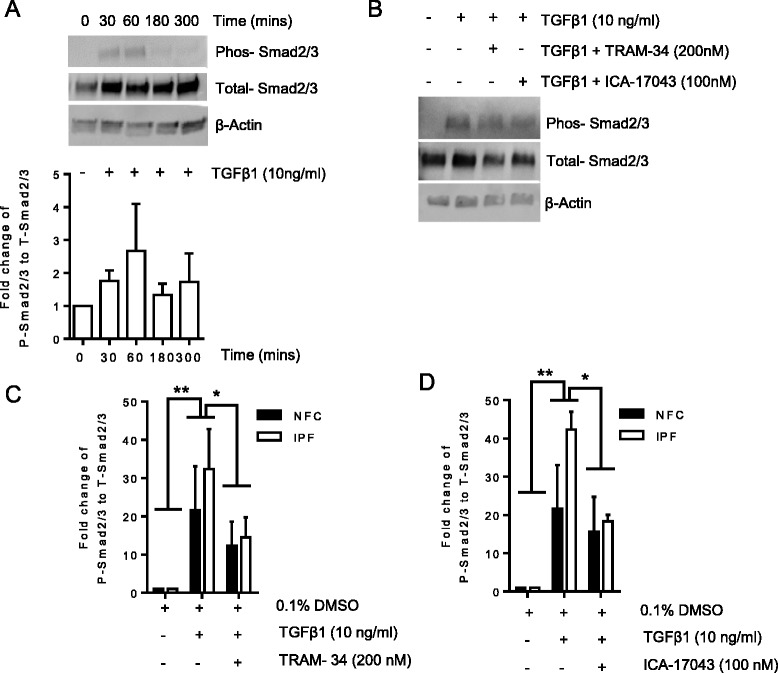
**Phosphorylation of Smad2/3 is K**_**Ca**_ **3.1 dependent. (A)** Phosphorylation of Smad2/3 in HLMFs was most abundant after 60 min of stimulation with TGFβ1 (10 ng/ml) (*n* = 3). **(B)** Representative Western blot analysis showing the increased phosphorylation of Smad2/3 after 60 min of stimulation with TGFβ1 (10 ng/ml) and its inhibition by TRAM-34 and ICA-17043. Phosphorylation of Smad2/3 was examined by fold change over total Smad2/3 and normalized to β-actin. TGFβ1-dependent increases in phosphorylated Smad2/3 were inhibited by TRAM-34 200 nM (NFC *n* = 3 and IPF *n* = 4) **(C)** and by ICA-17043 100 nM (NFC *n* = 3 and IPF *n* = 3) **(D)**. Results are represented as mean ± SEM *P < 0.05, **P < 0.01 (repeated measures ANOVA corrected by Sidaks multiple comparison test).

The ability of K_Ca_3.1 blockers to attenuate several TGFβ1-dependent cell processes in HLMFs [29] suggests that K_Ca_3.1 potentially regulates the activity of TGFβ1-dependent transcription factors. Smad2 and Smad3 are implicated as the major factors regulating HLMF differentiation and collagen secretion in response to TGFβ1 [20,21,31]. Phosphorylation is a key initial event in the activation of these Smad proteins. Using Western blot analysis, we therefore investigated the effect of TGFβ1 (10 ng/ml) on the phosphorylation of Smad2/3 and the expression of total Smad2/3 in HLMFs. Phosphorylation of Smad2/3 after TGFβ1 stimulation was examined over a time course of 5 h and peaked at 60 min (Figure 1A). No significant differences in Smad2/3 phosphorylation were seen between non-fibrotic controls (NFC) and IPF data, and data were therefore pooled for statistical analysis here and elsewhere where no disease-related differences were evident. Further experiments were therefore performed after 60-min stimulation with TGFβ1 in the presence or absence of the highly selective K_Ca_3.1 blockers TRAM-34 (Kd 20 nM) [24] and ICA-17043 (Kd 10 nM) [32]. TGFβ1 increased Smad2/3 phosphorylation in both NFC and IPF-derived HLMFs (*P* = 0.0022, paired *t* test). This was suppressed by both TRAM-34 and ICA-17043 (*P* = 0.0117 and *P* = 0.0144, respectively, one-way ANOVA corrected by Sidaks multiple comparisons test) (Figure 1B, C, D).

### TGFβ1-dependent Smad2/3 nuclear translocation is disrupted by K_Ca_3.1 inhibition

**Figure 2:**
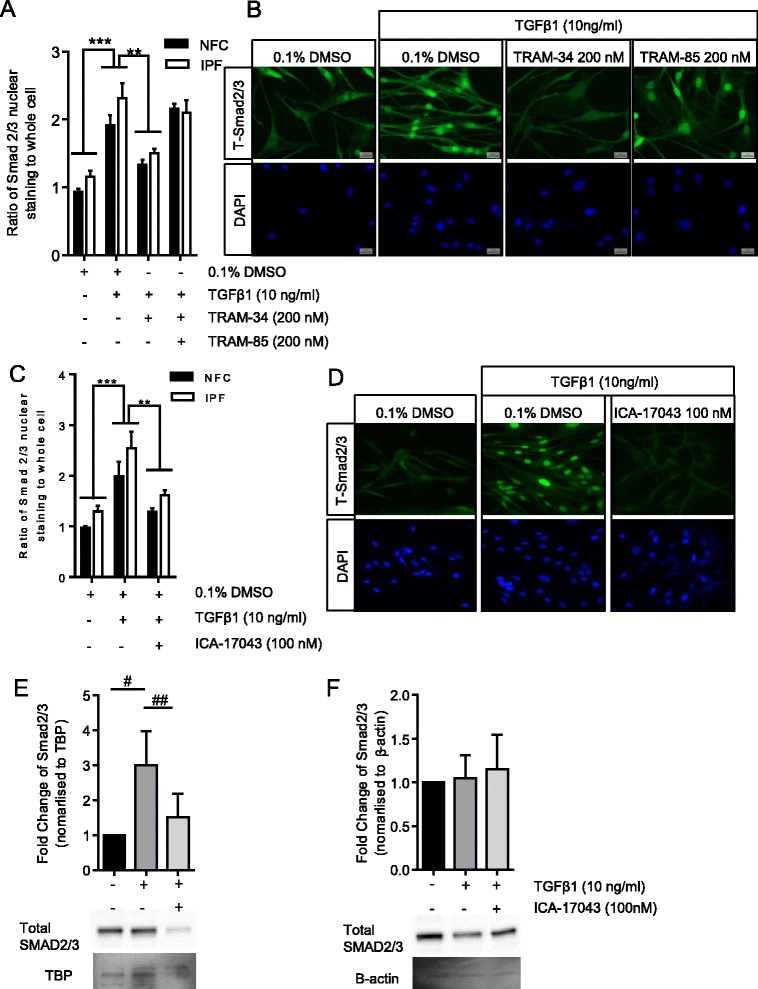
**Smad2/3 nuclear translocation is inhibited by K**_**Ca**_ **3.1 channel blockade. (A)** The ratio of nuclear to whole cell staining of Smad2/3 demonstrates a significant increase in nuclear translocation following TGFβ1 stimulation compared to control. TRAM-34 (200 nM) inhibited TGFβ1-induced Smad2/3 nuclear translocation (NFC *n* = 4, IPF *n* = 4) whereas the structurally related molecule TRAM-85 without K_Ca_3.1 blocking properties did not inhibit nuclear translocation. NFC and IPF data were pooled for statistical analysis. **(B)** Representative fluorescent microscopy images illustrate the increased expression of Smad2/3 following TGFβ1 stimulation and its movement into the nucleus, which was significantly attenuated by TRAM-34 (200 nM). **(C)** ICA-17043 (100 nM) also significantly attenuated Smad2/3 nuclear translocation, which can be seen visually in **(D). (E)** Quantification of Western blot analysis confirms that the total Smad2/3 in the nuclear enriched fraction is significantly increased following TGFβ1 stimulation and attenuated by ICA-17043 (100 nM). Results are normalized to TATA Binding Protein (TBP) and representative Western blot analysis images are shown. **(F)** Similarly, the total Smad2/3 was examined in the cytoplasmic-enriched fraction; however, no changes were found following TGβ1 stimulation or treatment with ICA-17043 (NFC *n* = 2 and IPF *n* = 3, data pooled). Results are represented as mean ± SEM ***P < 0.001, **P < 0.01 (two-way ANOVA corrected by Sidaks multiple comparison test), ^##^ *P* < 0.0001, one sample *t* test, ^#^ *P* < 0.05, paired *t* test.

Once TGFβ1 has induced phosphorylation of Smad2/3, this complex interacts with Smad4, which acts as a transporter and enters the nucleus to initiate gene transcription. Inhibition of Smad2/3 phosphorylation should therefore also prevent Smad2/3 nuclear translocation. We previously demonstrated using immunofluorescence that total Smad2/3 nuclear staining was significantly greater in IPF-derived HLMFs in comparison to NFC-derived cells [30], a finding also confirmed in this study (*P* = 0.0365). Here, TGFβ1 significantly increased Smad2/3 nuclear translocation in HLMFs over untreated control cells (*P* = 0.0025, paired *t* test) (Figure 2A, B, C, D). No significant differences were found between TGFβ1-stimulated NFC and IPF HLMF responses, and therefore, statistics were performed on pooled data. TRAM-34 (200 nM) or ICA-17043 (100 nM) significantly attenuated the nuclear translocation of Smad2/3 (*P* = 0.0034 and *P* = 0.0029, respectively, two-way ANOVA corrected by Sidak’s multiple comparison test). In contrast, the structurally related molecule, TRAM-85, which does not have channel blocking activity, did not inhibit nuclear translocation of Smad2/3 (Figure 2A, B, C, D). Total Smad2/3 was examined in the nuclear fraction and cytoplasmic extract of both NFC and IPF-derived HMFs. Following TGFβ1-stimulation, there was significantly greater amounts of total Smad2/3 located within the nucleus of HLMFs (*P* < 0.0001); furthermore, after inhibition with ICA-17043 (100 nM), this increase was attenuated (*P* = 0.0168, Figure 2E). No significant changes were found in the cytoplasmic extract (Figure 2F). Thus, K_Ca_3.1 activity is critical for the TGFβ1-dependent phosphorylation and nuclear translocation of Smad2/3.

### TGFβ1-dependent Smad2/3 phosphorylation and nuclear translocation are Ca^2+^-dependent

**Figure 3:**
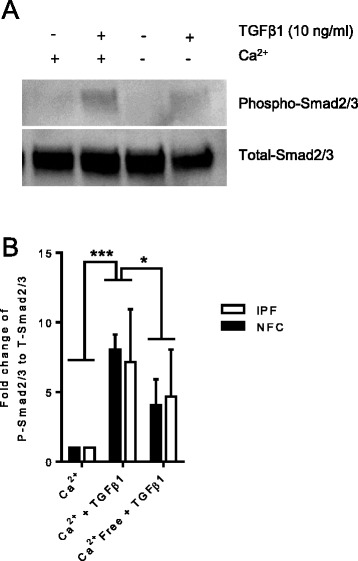
**TGF 1-dependent Smad2/3 phosphorylation is Ca** ^**2+**^ **dependent. (A)** A representative Western blot demonstrating Smad 2/3 phosphorylation when cells are incubated in media either containing Ca^2+^ or without. **(B)**. Quantification of Western blots showing that the increased phosphorylation of Smad2/3 after 60 min of TGFβ1 exposure (10 ng/ml) is largely dependent on the presence of extracellular Ca^2+^. Phosphorylation of Smad2/3 was examined by fold change over total Smad2/3 and normalized to β-actin (NFC *n* = 2, IPF *n* = 4, data pooled for statistical analysis). HLMFs stimulated for 1 h in Ca^2+^-free media phosphorylated significantly less Smad2/3 than those incubated in media containing Ca^2+^. *P < 0.05, ***P < 0.001 results are represented as median (IQR) (one-way ANOVA, corrected by Sidaks multiple comparison test).

**Figure 4:**
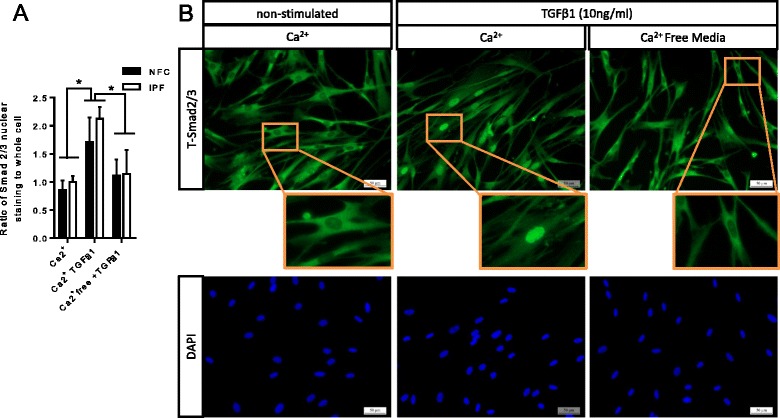
**TGFβ1-dependent Smad2/3 nuclear translocation is Ca** ^**2+**^ **dependent. (A)** The ratio of nuclear to whole cell TGFβ1-induced Smad 2/3 nuclear translocation is significantly attenuated when cells are incubated in media without Ca^2+^ (NFC *n* = 3, IPF *n* = 3, data pooled). **(B)** Fluorescent microscopy images illustrating the increased nuclear translocation of total Smad2/3 following TGFβ1 stimulation and its movement into the nucleus, which was significantly attenuated in the absence of Ca^2+^. Results are represented as mean ± SEM *P < 0.001 (repeated measures ANOVA corrected by Sidaks multiple comparison test).

We have shown previously that K_Ca_3.1 channel blockade inhibits a rise in intracellular Ca^2+^ in HLMFs following TGFβ1 stimulation [29]. This is likely due to plasma membrane depolarization which reduces Ca^2+^ entry [25,29]. If this is mechanistically important, lowering extracellular Ca^2+^ should also inhibit TGFβ1-dependent Smad2/3 phosphorylation and nuclear translocation. Myofibroblasts incubated for 1 h in Ca^2+^-free media phosphorylated significantly less Smad2/3 in the presence of TGFβ1 (10 ng/ml) than those incubated in media containing normal external Ca^2+^, *P* = 0.0439 (one-way ANOVA, corrected by Sidaks multiple comparison test) (Figure 3A, B). This was accompanied by reduced TGFβ1-dependent Smad2/3 nuclear translocation in Ca^2+^-free media, *P* = 0.0114 (Figure 4A, B). This suggests that the enhancement of Ca^2+^-influx by K_Ca_3.1 channels is an essential requirement for the efficient TGFβ1-dependent phosphorylation and nuclear translocation of Smad2/3.

### TGFβ1-dependent increases in αSMA, collagen type I and K_Ca_3.1 mRNA are inhibited following K_Ca_3.1 channel block

**Figure 5:**
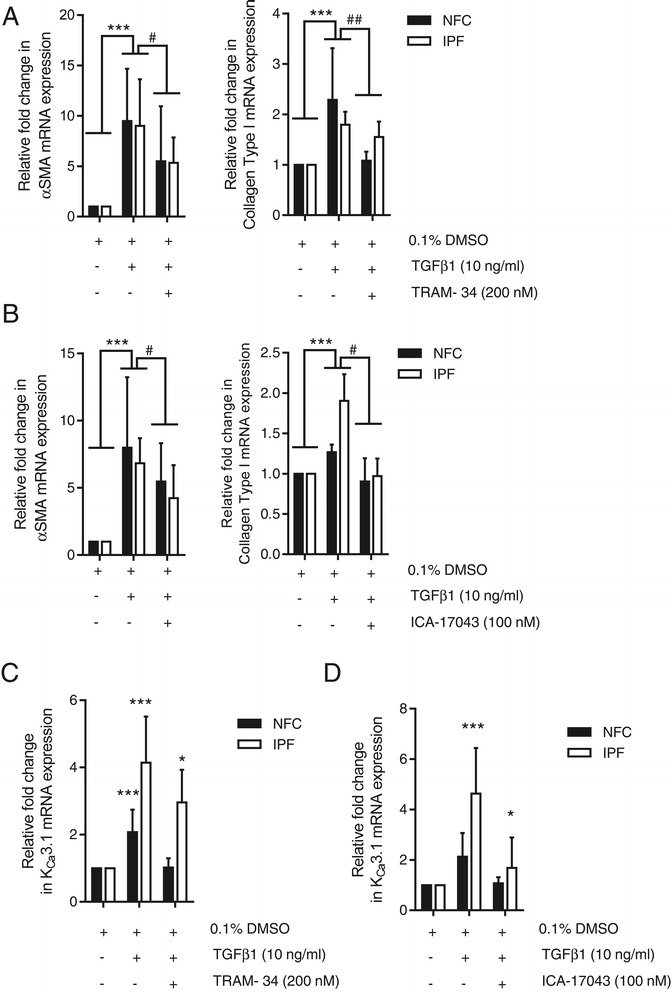
**TGFβ1-dependent transcription of αSMA, collagen type I and K**_**Ca**_ **3.1 is K**_**Ca**_ **3.1 dependent. (A)** TGFβ1 stimulation significantly increased αSMA and collagen I mRNA expression in HLMFs per 10^3^ copies of β-Actin, which was significantly inhibited by TRAM-34 200 nM (NFC *n* = 4, IPF *n* = 4, data pooled for statistical analysis). **(B)** Similarly, ICA-17043 (100 nM) also significantly decreased TGFβ1-dependent increases in αSMA and collagen I mRNA expression in HLMFs (NFC *n* = 3, IPF *n* = 3, data pooled). Results are represented as mean ± SEM or median (IQR) ***P < 0.001 (one sample *t* test), ^#^ *P* < 0.05 and ^##^ *P* < 0.01 (paired *t* test or Wilcoxon signed rank test). **(C, D)** The fold change of TGFβ1-dependent K_Ca_3.1 mRNA expression was significantly higher in IPF HLMFs compared to that in NFC HLMFs, ^#^ *P* = 0.0313 (NFC *n* = 4, IPF *n* = 4, data pooled for statistical analysis). This TGFβ1 dependent increase in K_Ca_3.1 mRNA in IPF donors was significantly attenuated by K_Ca_3.1 channel blockers, TRAM-34 and ICA-17043 (*P = 0.0385 and *P* = 0.0313, respectively, paired *t* test).

Impaired TGFβ1-dependent Smad2/3 nuclear translocation with K_Ca_3.1 blockade predicts that TGFβ1-dependent gene transcription will also be inhibited. To assess this, we first undertook quantitative RT-PCR to examine the mRNA expression of αSMA and collagen type I in HLMFs. TGFβ1 (10 ng/ml) stimulation for 24 h upregulated both αSMA and collagen type I mRNA expression, and these increases were significantly inhibited by both TRAM-34 (200 nM) and ICA-17043 (100 nM) (Figure 5A, B).

K_Ca_3.1 mRNA and functional channel expression are also upregulated in HLMFs by TGFβ1 [29]. We were therefore interested in whether K_Ca_3.1 regulates its own mRNA expression. We found that TGFβ1 also significantly increased K_Ca_3.1 mRNA expression within HLMFs, and this upregulation was significantly higher in IPF HLMFs in comparison to NFC HLMFs, as previously published [29]. This increase was also inhibited by the K_Ca_3.1 channel blockers TRAM-34, and ICA-17043 (*P* = 0.0385 and *P* = 0.0313, respectively, Wilcoxon-matched pairs rank test) (Figure 5C, D). Thus, K_Ca_3.1 channel block inhibited the transcription of several diverse TGFβ1-regulated genes (αSMA - structural protein, collagen type I - secreted matrix protein, K_Ca_3.1 - ion channel), in keeping with its ability to inhibit Smad2/3 phosphorylation and nuclear translocation.

### K_Ca_3.1 block attenuates TGFβ1-induced increases in αSMA protein expression

We and others have shown previously that IPF-derived HLMFs express higher levels of αSMA than NFC HLMFs constitutively, and we showed that this is inhibited by K_Ca_3.1 blockade. Because TGFβ1-dependent transcription of the αSMA gene is also inhibited by K_Ca_3.1 blockers, we proceeded to investigate the effect of K_Ca_3.1 blockers on TGFβ1-dependent αSMA protein expression in HLMFs.

**Figure 6:**
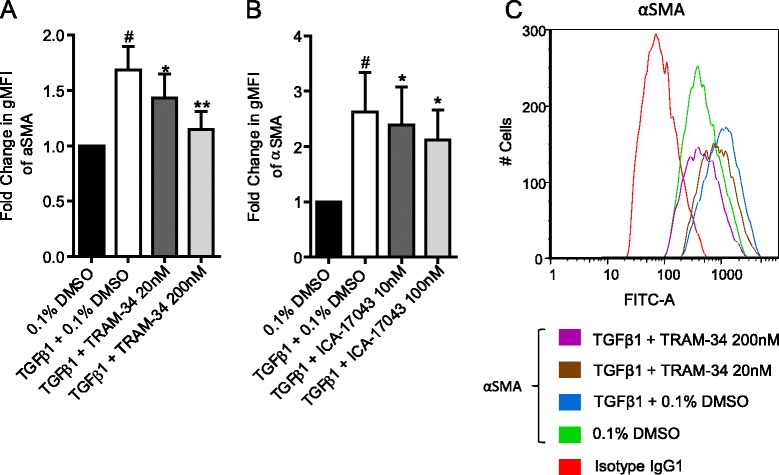
**TGFβ1-dependent αSMA protein expression is attenuated by K**_**Ca**_ **3.1 channel block. (A)** Flow cytometry measurements of αSMA expression in HLMFs show that the fold change in gMFI was significantly increased by TGFβ1 (10 ng/ml) (NFC *n* = 3, IPF *n* = 4, data pooled for statistical analysis). TRAM-34 (20 and 200nM) dose-dependently decreased TGFβ1-induced αSMA expression. **(B)** Similarly, ICA-17043 (10 and 100nM) significantly reduced TGFβ1-induced αSMA expression. **(C)** Representative fluorescent histogram showing αSMA expression in HLMFs under the above conditions. Results are represented as mean ± SEM. ^#^ *P* < 0.05 (paired *t* test); *P < 0.05, **P < 0.01 (corrected by Dunn’s multiple comparisons test).

Flow cytometry was used to examine αSMA expression in HLMFs following 24 h of TGFβ1-stimulation in the presence of K_Ca_3.1 channel blockers. TGFβ1 significantly increased αSMA protein expression in both NFC- and IPF-derived myofibroblasts (*P* = 0.0156, Wilcoxon signed rank test). TRAM-34 (20 and 200 nM) and ICA-17043 (10 and 100 nM) both dose-dependently decreased TGFβ1-dependent αSMA expression when examined by the fold change in geometric mean fluorescent intensity, *P* = 0.0052 and *P* = 0.0278, respectively (one-way ANOVA) (Figure 6A, B, C). This indicates a phenotypic transition of HLMFs into a more fibroblast-like phenotype, which would explain the reduced contraction, secretion and proliferative abilities demonstrated in previously published work [29].

## Discussion

This study demonstrates that Ca^2+^ and the Ca^2+^-activated K^+^ channel K_Ca_3.1 play a key role in the TGFβ1-dependent activation of Smad2/3 in HLMFs. Inhibition of K_Ca_3.1 channels therefore attenuates TGFβ1-dependent gene transcription, resulting in the inhibition of pro-fibrotic HLMF activity [30] and de-differentiation of HLMFs towards a fibroblast phenotype as indicated by a reduction in αSMA protein.

This paper provides mechanistic insight into the inhibitory effects of K_Ca_3.1 blockers on HLMF pro-fibrotic function. Open K^+^ channels hyperpolarize the plasma membrane and increase Ca^2+^ entry following receptor-dependent cell activation [29,33]. We have demonstrated previously that blocking K_Ca_3.1 channels reduces constitutive αSMA expression and Smad2/3 nuclear localization in NFC- and IPF-derived HLMFs, although it was not possible to detect Smad2/3 phosphorylation under these conditions [30]. We have also shown previously that K_Ca_3.1 activity is required for a rise in intracellular Ca^2+^ that occurs following exposure of HLMFs to TGFβ1 [29]. Importantly, here, we have shown that extracellular Ca^2+^ and K_Ca_3.1 activity are required for the TGFβ1-dependent phosphorylation and nuclear translocation of Smad2/3, an essential initial step in Smad2/3 activation. Thus, there is a clear mechanistic link between K_Ca_3.1 activity and Smad2/3-dependent cell signalling. Although other signalling cascades including ras/MEK/ERK and P38 MAPK are also involved in TGFβ1 pro-fibrotic signalling, our data nevertheless suggest that the predominant regulation of the TGFβ1 signalling pathway by K_Ca_3.1 channels occurs proximally at or above the level of Smad2/3 phosphorylation, via effects on Ca^2+^ signalling.

This study both supports and extends the work of others investigating the role of K_Ca_3.1 in renal fibrosis [34], liver fibrosis [35] and brain pathology [36]. Murine renal fibroblast proliferation and kidney fibrosis following ureteric obstruction were significantly attenuated by K_Ca_3.1 blockers [37], while in a streptazocin mouse model of diabetes-induced renal fibrosis, K_Ca_3.1 inhibition reduced TGFβ1, TGFBRII and phosphoSmad2/3 expression in the tissue [34,38]. Subsequent work from the same authors using human renal interstitial fibroblasts showed that TGFβ1-dependent increases in types I and IV collagen and αSMA mRNA expression were attenuated by K_Ca_3.1 blockade, although there was only a reduction in type IV collagen protein expression reported [34]. In contrast, we found clear evidence of reduced collagen type I mRNA expression in keeping with previous observations that K_Ca_3.1 blockade attenuates TGFβ1-dependent HLMF collagen secretion [29] and that fibroblasts lacking the Smad3 gene have reduced induction of collagen type I gene [39]. We also found that TGFβ1-dependent αSMA gene transcription was inhibited by K_Ca_3.1 blockers, and TGFβ1-dependent HLMF αSMA protein expression were reduced. In keeping with the work of Huang *et al*., we also found that TGFβ1-dependent phosphorylation of Smad2/3 was attenuated by K_Ca_3.1 blockade [38], but in addition show that this prevents Smad2/3 nuclear translocation.

It is important that we have demonstrated the effects of K_Ca_3.1 inhibition in parenchymal HLMFs because of the functional heterogeneity evident in fibroblasts from different tissues and between species [40-42]. Moreover, in previous studies of renal fibroblasts, the concentration of TRAM-34 (2 μM) used was relatively high [38]. We used two distinct K_Ca_3.1 blockers at the IC_50_ (20 nM for TRAM-34 and 10 nM for ICA-17043) and at 10× the IC_50_ where >95% of channels will be blocked and found clear evidence for activity of these drugs at these physiologically relevant concentrations. Importantly, the concentration of ICA-17043 used here can be achieved *in vivo* with oral dosing [43], although the free drug concentration will be reduced due to protein binding. Thus, pharmacological activity is likely to be achieved within human lung following oral administration.

We found previously that K_Ca_3.1 inhibition reduced TGFβ1-dependent HLMF contractility in collagen gels [29]. Our demonstration that this is associated with reduced αSMA gene transcription and protein expression suggests that de-differentiation towards a fibroblast phenotype with reduced contractile machinery may explain this reduced contractility, at least in part. However, attenuation of Ca^2+^ influx as demonstrated previously [29], with depolarization of the plasma membrane could also contribute to the effects of K_Ca_3.1 blockade on HLMF contraction.

Our findings also provide a potential mechanism for the ability of K_Ca_3.1 inhibition to prevent airway wall remodelling in a mouse model of asthma [44]. That study demonstrated that treatment with TRAM-34 reduced airway smooth muscle mass, peribronchial fibrosis, and sub-epithelial collagen expression. While these effects might have occurred in part through the inhibition of the associated inflammatory response, it seems likely that a direct effect on airway smooth muscle and myofibroblast activity was also relevant.

## Conclusions

**Figure 7:**
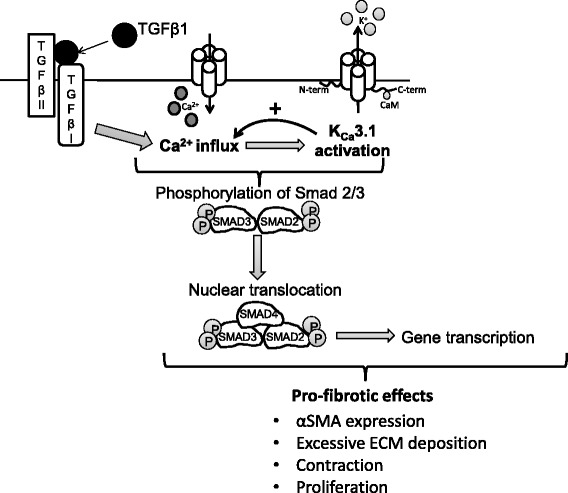
**The involvement of K**_**Ca**_ **3.1 in the TGF/Smad 2/3 signalling pathway.** A diagrammatic representation of how Smad2/3 phosphorylation and subsequent nuclear translocation is reliant on an influx of extracellular Ca^2+^ and K_Ca_3.1 ion channels. TGFβ1 stimulation triggers an influx of extracellular Ca^2+^, which in turns opens Ca^2+^-activated K_Ca_3.1 K^+^ channels. K_Ca_3.1 opening maintains a negative membrane which in turn promotes Ca^2+^ entry. Phosphorylation of Smad2/3 and therefore its downstream effects such as translocation and gene transcription are heavily reliant on Ca^2+^. K_Ca_3.1 channel inhibition reduces Ca^2+^ entry, which in turn reduces Smad2/3 phosphorylation and nuclear translocation, and thus reduces TGFβ1-dependent gene transcription.

Myofibroblast activity is clearly a key driver of tissue fibrosis and shrinkage in IPF [45-47]. There is strong evidence that TGFβ1-dependent signalling contributes to this fibrotic process [12,14,20,30,38,48]. However, there is also evidence that HLMFs derived from IPF lungs also exhibit greater constitutive pro-fibrotic activity, which might occur due to pre-programming by genetics, and/or re-programming by epigenetics [30,49]. Importantly, K_Ca_3.1 blockade is able to inhibit both TGFβ1-dependent and constitutive pro-fibrotic HLMF activity as shown here and in our previous study [30], respectively. This provides further support for the view that K_Ca_3.1 may provide a novel and effective target for the treatment of IPF. Furthermore, the capacity for K_Ca_3.1 inhibition to downregulate TGFβ1-dependent increases in K_Ca_3.1 expression suggests that inhibiting K_Ca_3.1 will rapidly downregulate the TGFβ1 axis through positive feedback (Figure 7). K_Ca_3.1 knockout animals are relatively healthy, and an oral K_Ca_3.1 inhibitor, ICA-17043 (Senicapoc), was well tolerated for 12 months in a phase III clinical trial of sickle cell disease [43]. There is therefore the potential for the rapid translation of K_Ca_3.1-directed therapy to the clinic.

## Methods

### Ethics statement

All patients donating tissue gave written informed consent, and the study was approved by the National Research Ethics Service (references 07/MRE08/42 and 10/H0402/12).

### Human lung myofibroblasts isolation and culture

NFC parenchymal HLMFs were derived from healthy areas of lung from patients undergoing lung resection for carcinoma at Glenfield Hospital, Leicester, UK. No morphological evidence of disease was found in the tissue samples used for HLMF isolation. IPF HLMFs were derived from patients undergoing lung biopsy for diagnostic purposes at the University of Pittsburgh Medical Center, USA, and were shown to have UIP on histological examination. HLMFs were grown from explanted lung tissue from both sources under identical conditions, using Dulbecco’s modified Eagle’s medium (DMEM) supplemented with 10% fetal bovine serum (FBS), antibiotic/antimycotic agents and non-essential amino acids [50,51]. The cells were cultured at 37°C in 5% CO_2_/95% air. The cells were studied at passages 4 to 5 for functional studies. Myofibroblasts were characterized as previously described [29]. All NFC patients gave informed written consent, and the study was approved by the Leicestershire, Northamptonshire and Rutland Research Ethics Committee 2. Written informed consent was also obtained from all IPF subjects, in accordance with the responsible University of Pittsburgh Institutional Review Board.

### Flow cytometry

HLMFs were grown on T25 flasks and serum starved for 24 h prior to the experiment. The HLMFs were either left unstimulated or stimulated for 24 h with TGFβ1 (10 ng/ml), in the presence of 0.1% DMSO control, TRAM-34 (200 nM) or ICA-17043 (100 nM). The cells were detached using 0.1% trypsin/0.1% EDTA, washed then fixed and permeabilized in 4% paraformaldehyde plus 0.1% saponin (Sigma-Aldrich, St. Louis, MO, USA), respectively, for 20 min on ice. Myofibroblasts were labelled with FITC-conjugated mouse monoclonal anti-αSMA (Sigma-Aldrich, St. Louis, MO, USA), indirectly labelled with FITC and isotype control FITC-conjugated mouse IgG_2a_. Secondary antibodies labelled with FITC (F0313, Dako, Glostrup, Denmark) were applied. Analysis was performed using single colour flow cytometry on a FACScan (BD, Oxford, UK).

### qRT-PCR

HLMF RNA was isolated using the RNeasy Plus Kit (Qiagen, West Sussex, UK) according to the manufacturer’s instructions. Primers were designed for ACT2A, forward TTCAATGTCCCAGCCATGTA and reverse GAAGGAATAGCCACGCTCAG, product size 222 bp from NCB1 Reference sequence NM_001141945.1 and COL1A1, forward TTCTGCAACATGGAGACTGG and reverse CGCCATACTCGAACTGGAATC, product size 151 bp from reference sequence NM_000088.3. K_Ca_3.1 and β-actin primers were analysed using gene-specific Quantitect Primer Assay primers (Qiagen, Hilden, Germany), Hs_KCNN4_1_SG and HS_ACTB_1_SG. All experiments were performed in duplicate. All expression data was normalized to β-actin and corrected using the reference dye ROX. Gene expression was quantified by real-time PCR using the Brilliant SYBR Green QRT-PCR 1-Step Master Mix (Stratagene, Breda, The Netherlands). PCR products were run on a 1.5% agarose gel to confirm the product amplified was the correct size, and they were sequenced to confirm the specificity of the primers. Prior to qRT-PCR, myofibroblasts were grown to confluence, serum starved for 24 h and then stimulated with TGFβ1 (10 ng/ml) in the presence of 0.1% DMSO control, TRAM-34 (200 nM) or ICA-17043 (100 nM).

### Western blot for SMAD proteins

Cells were grown in T75 flasks, serum starved for 24 h and stimulated with TGFβ1 (10 ng/ml) in the presence of either 0.1% DMSO control, TRAM-34 (200 nM), ICA-17043 (100 nM) or Ca^2+^-free media for 1 h. The cells were detached with 0.1% Trypsin/EDTA and washed. The protein was isolated using RIPA buffer lysis system (Santa Cruz, Heidelberg, Germany), and the total protein concentration was determined using the DC Bio-Rad protein Assay (Bio-Rad, Hemel Hempstead, UK). Of the protein, 30 μg was resolved using 10% Mini-Protean TGX precast gels (Bio-Rad, Hemel Hempstead, UK) and then transferred to an immunobilon-P polyvinylidene difluoride membrane, using Trans-blot Turbo transfer packs (Bio-Rad, Hemel, Hemopstead, UK). The membranes were blocked with 5% milk and incubated with rabbit monoclonal anti-phospho-Smad2/Smad3 (0.231 μg/ml, Cell Signalling, Danvers, MA, USA) or rabbit monoclonal anti-smad2/smad3 (0.0087 μg/ml, Cell Signalling). Protein bands were identified by horseradish peroxidase-conjugated secondary antibody and enhanced chemiluminescence reagent (Amersham Laboratories, Buckinghamshire, UK). Immunolabelled proteins were visualized using ImageQuant LAS 4000 (GE Healthcare Life Sciences, Buckinghamshire, UK).

### SMAD nuclear translocation

HLMFs were grown on 8-well chamber slides and serum-starved for 24 h prior to the experiment. The cells were then stimulated with TGFβ1 (10 ng/ml) in the presence of either 0.1% DMSO control, TRAM-34 (200 nM), ICA-17043 (100 nM), TRAM-85 (200nM) or Ca^2+^-free media. After 1 h, the cells were fixed with methanol for 20 min on ice, blocked using 3% BSA for 1 h and immunostained using rabbit monoclonal anti-Smad2/Smad3 (0.174 μg/ml, Cell Signalling). Secondary antibody labelled with FITC (F0313, Dako) was applied, and the cells counterstained with 4′,6-diamidino-2-phenylindole (DAPI, Sigma-Aldrich, St. Louis, MO, USA). The cells were mounted with fluorescent mounting medium and cover-slipped.

Original images were captured on an epifluorescent microscope (Olympus BX50, Olympus UK Ltd., Southend-on-sea, Essex, UK); grey scale intensity was examined using Cell F imaging software (Olympus UK Ltd., Essex, UK). Matched exposures were used for isotype controls. The intensity of nuclear Smad2/3 staining was quantified by measuring the grey scale intensity of DAPI positive nuclei with a minimum of 10 random cells measured in one field for each condition.

### Nuclear fraction

NFC and IPF-derived HLMFs were grown in T75 flasks, serum-starved for 24 h then stimulated with TGFβ1 (10 ng/ml) in the presence of either 0.1% DMSO control, TRAM-34 (200 nM) or ICA-17043 (100 nM). After 1 h, HLMF were detached with 0.1% Trypsin/EDTA and washed. Nuclear and cytoplasmic extracts were isolated using the Nuclear Extract Kit (Abcam, ab113474, New territories, Hong Kong). Proteins of both the nuclear and cytoplasmic extracts were then isolated using the RIPA buffer lysis system and the concentration determined using the DC Bio-Rad protein Assay. Western blot was then performed as described above.

### Statistical analysis

Experiments from an individual donor were performed either in duplicate or triplicate, and a mean value was derived for each condition. Data distribution across donors was tested for normality using the Kolmogorov-Smirnov test. For parametric data, the one-way ANOVA or repeated measures ANOVA for across-group comparisons was used followed by the appropriate multiple comparison *post hoc* test; otherwise, an unpaired or paired *t* test was used. Where appropriate, a one sample *t* test was used using a null hypothesis of 1. For non-parametric data, the Friedman test was used for across group comparisons followed by the appropriate multiple comparison *post hoc* test, or the Mann Whitney *U* test was used where there were two unpaired groups. GraphPad Prism for windows (version 6, GraphPad Software, San Diego, CA, USA) was used for these analyses. A value of *P* < 0.05 was taken to assume statistical significance and data are represented as mean (± SEM) or median (IQR).

## Abbreviations

DMEM: Dulbecco’s modified eagle’s medium
FBS: Fetal bovine serum
HLMF: Human lung myofibroblast
IPF: Idiopathic pulmonary fibrosis
IQR: Interquartile range
NFC: Non-fibrotic control
SEM: Standard error of the mean
TGFβ1: Transforming growth factor beta 1
TGFβRII: Transforming growth factor beta receptor II
αSMA: α-smooth muscle actin
